# Late Diagnosis of Kartagener Syndrome in an Adult Female

**DOI:** 10.7759/cureus.58747

**Published:** 2024-04-22

**Authors:** Ilianna Tsetsou, Vasileios Balomenos, Panagiotis Koreas, Irini Elissavet Biliara, Kyriaki Tavernaraki

**Affiliations:** 1 Department of Imaging and Interventional Radiology, “Sotiria” General and Chest Diseases Hospital of Athens, Athens, GRC

**Keywords:** kartagener syndrome, chronic sinusitis, bronchiectasis, situs inversus, primary ciliary dyskinesia

## Abstract

Kartagener syndrome (KS), also known as primary ciliary dyskinesia, is a rare genetic disorder commonly diagnosed early in childhood. It is characterized by a triad of findings, namely, situs inversus, chronic sinusitis, and bronchiectasis. Here, we present the case of a 73-year-old female who incidentally presented the KS triad during her imaging tests in the emergency department of our institution for COVID-19 symptoms.

## Introduction

Kartagener syndrome (KS), also known as primary ciliary dyskinesia (PCD), is a rare genetic disorder that primarily affects the function of cilia in the respiratory and reproductive systems [1]. KS is characterized by a triad of symptoms: situs inversus, where the major internal organs, such as the heart and liver, are mirrored or reversed from their normal positions, bronchiectasis, and chronic sinusitis [2].

Chronic respiratory problems are caused by the decreased motility of cilia and thus mucus and debris are trapped in the respiratory tract (RT). Common respiratory symptoms include chronic cough, recurrent respiratory infections (e.g., sinusitis and bronchitis), and wheezing. Defective cilia in the reproductive tract can lead to sperm immobility in males, whereas ciliary dysfunction can impair the movement of follicles within the fallopian tubes in females, increasing the risk of infertility [3]. Additional characteristics and features of KS may include nasal congestion and sinusitis, ear infections, heterotaxy, and cardiovascular anomalies [1,4].

KS is an autosomal recessive genetic disorder and the genetic mutations associated with it affect the structure and function of the dynein arms within the cilia, leading to impaired ciliary movement. The diagnosis of KS typically involves a combination of clinical evaluation, imaging studies (e.g., chest X-rays and CT scans), and specialized tests such as high-speed video microscopy to assess ciliary function. Genetic testing can confirm the presence of specific gene mutations associated with the condition [5].

This case report describes a 73-year-old female who incidentally presented the KS triad during her imaging tests in the emergency department (ED) of our institution for COVID-19 symptoms.

## Case presentation

**Figure 1:**
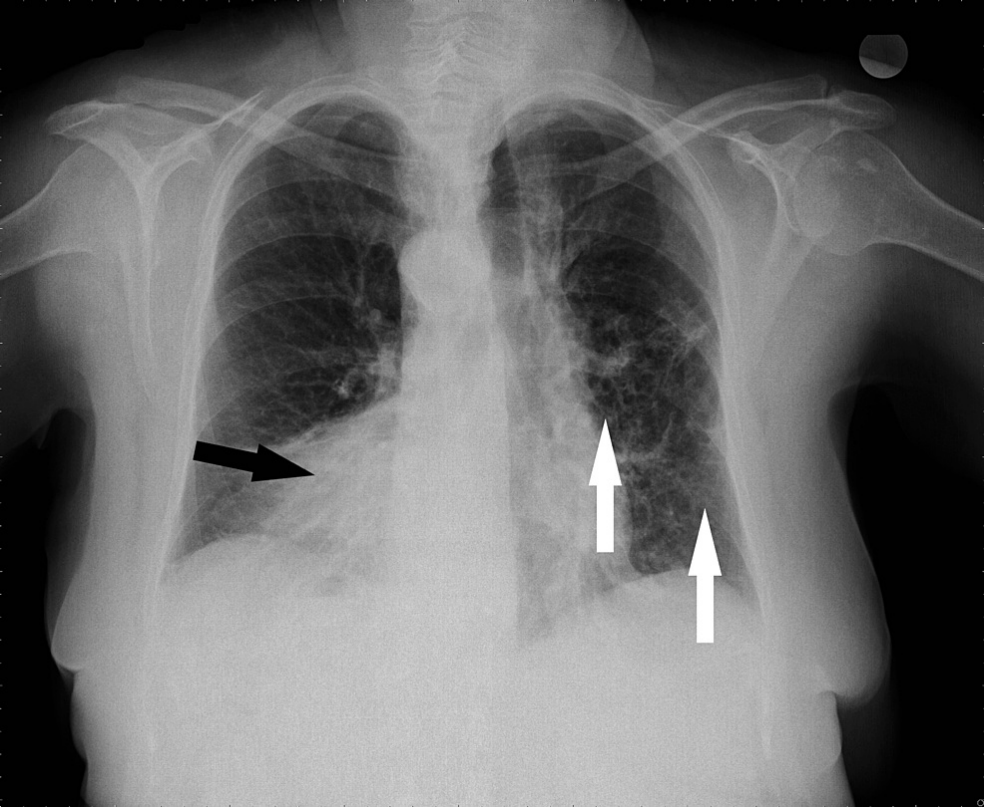
Chest X-ray. Chest X-ray in the emergency department showing the cardiac apex, the aortic arch, and stomach bubble on the right side, suggesting situs inversus. The black arrow indicates dextrocardia. There are confluent, cyst-like structures on the left lower lung zone, suggesting the presence of bronchiectases (white arrows).

A 73-year-old female was assessed in the ED of our institution for shortness of breath, productive cough, fever (38.5°C), and 90% oxygen saturation associated with COVID-19. A physical examination revealed nasal flaring, while chest auscultation and examination revealed diminished air movement. No other remarkable findings were noted. The patient reported at least two middle ear infections per year. Chest X-ray showed the cardiac apex, the aortic arch, and stomach air on the right side, suggesting situs inversus (Figure 1).

**Figure 2:**
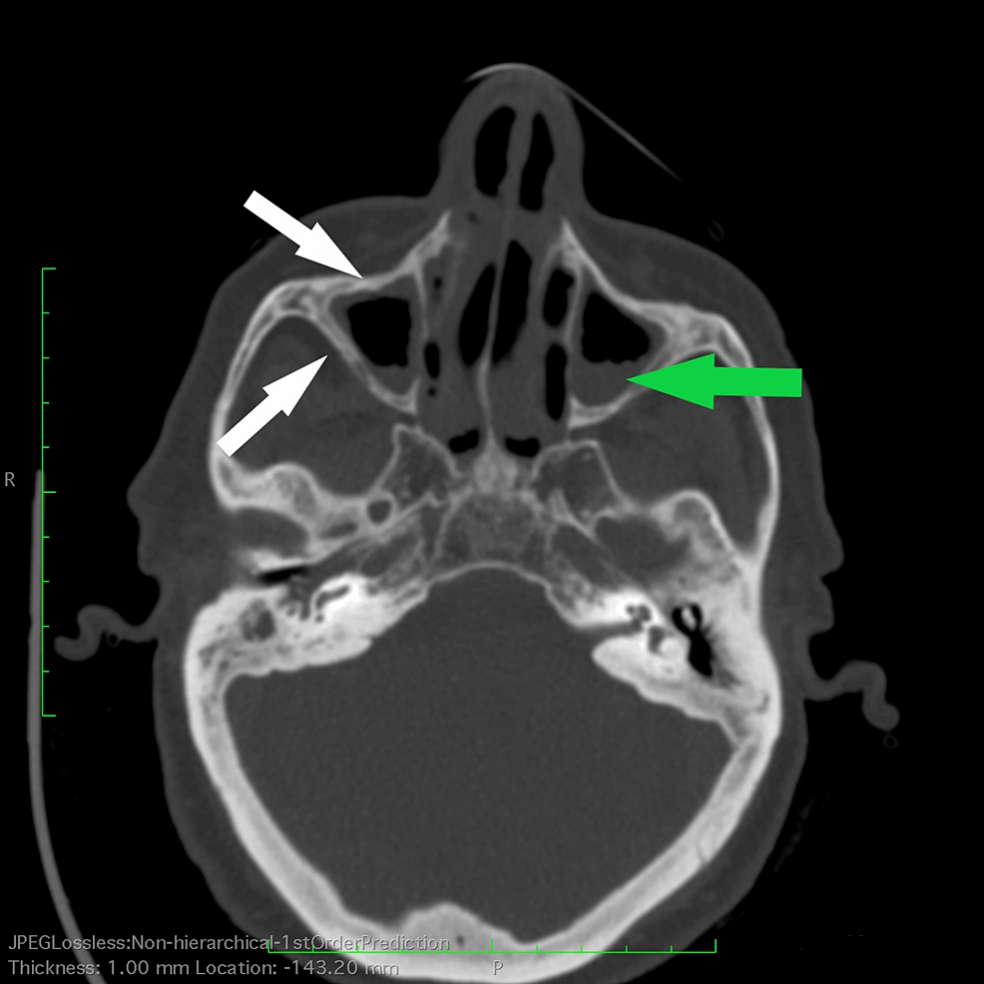
Sinus CT scan. Sinus CT scan (bone window) showing secretions and mucosal thickening (green arrow) in both maxillary sinuses as well as sclerotic thickened bone involving the sinus walls (white arrows), suggestive of acute and chronic sinusitis.

ED doctors ordered paranasal sinuses and chest CT scans to further evaluate the patient’s condition. A CT scan of the paranasal sinuses revealed secretions and mucosal thickening of the frontal, maxillary, and ethmoidal cavities, as well as sclerotic bone changes of the sinus walls, suggestive of active sinusitis and chronic changes (Figure 2).

**Figure 3:**
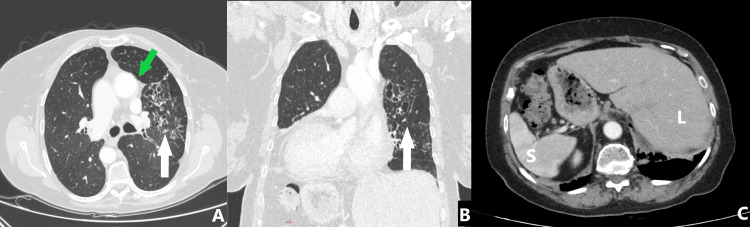
Chest CT. Chest CT (with intravenous contrast) showing (A) cystic bronchiectases mostly on the left upper lobe and lingula and diffuse mosaic attenuation (white arrows). The ascending aorta is located on the left (green arrow). No other cardiac anomalies are noted (not shown). (B) Dextrocardia and bronchiectasis (white arrows) on the left upper lobe. (C) Situs inversus. The liver (L) and spleen (S) are reversed.

A chest CT scan (Figure 3) depicted the situs inversus seen on the X-ray previously. An additional horizontal fissure was reported in the left lung. Moreover, cystic bronchiectasis in the left upper lobe as well as smaller in the right upper lobe and the middle lobe with wall enlargement were observed. Centrilobular micronodules were depicted in both lungs with small opacities near the final bronchioles. Small opacities were also reported at the inferior segments of the lower lobes as well as near the left hilum. Bands of atelectasis were seen in the middle lobe, whereas smaller ones were observed in the inferior surface of the lower lobes. The available images of the upper portion of the abdomen revealed mirror transposition of the liver and spleen.

The patient was then admitted to the internal medicine department’s COVID-19 wing for treatment. During her admission, PCD was officially established after the genetic tests confirmed the existence of the relevant mutations associated with the condition. After one week, the patient was released and follow-up imaging was scheduled on an outpatient basis.

## Discussion

KS was first described by Manes Kartagener in 1933 as a triad of situs inversus, chronic sinusitis, and bronchiectasis [1]. Later, in 1995, Bjorn Afzelius published a paper on men with sperm immotility disorders and situs inversus when he noticed a pattern, leading to further research on diseases of the cilia [2].

KS is part of the broader category of PCD, a rare, autosomal recessive genetic disorder of the cilia, causing defects in their normal function and mucous clearance [3]. The prevalence of PCD varies due to insufficient data and is estimated to be around 1:2,200 to 1:40,000 [6]. Common clinical manifestations of KS in children and adults are chronic upper RT infections, such as otitis media and rhinosinusitis, recurrent lower RT infections, such as bronchitis and pneumonia, usually leading to bronchiectasis formation, as well as infertility. Diagnosis is usually established during the neonatal period or childhood while investigating recurrent RT infections or situs inversus [4].

According to the PCD Foundation consensus recommendations, diagnosis of PCD/KS requires fulfillment of clinical, genetic, and laboratory criteria (Table 1) [5].

**Table 1:** PCD diagnostic criteria (by age). Diagnosis, monitoring, and treatment of primary ciliary dyskinesia: PCD foundation consensus recommendations based on the state-of-the-art review. Shapiro AJ, Zariwala MA, Ferkol T, et al.: Diagnosis, monitoring, and treatment of primary ciliary dyskinesia: PCD foundation consensus recommendations based on state of the art review. Pediatr Pulmonol. 2016, 51:115–32. 10.1002/ppul.23304 [5]. PCD: primary ciliary dyskinesia; CPAP: continuous positive airway pressure

PCD diagnostic criteria (by age)	
Newborns (0–1 month of age)	
Situs inversus totalis and unexplained neonatal respiratory distress at term birth	
Plus at least one of the following	Diagnostic ciliary ultrastructure on electron micrographs
	Biallelic mutations in one PCD-associated gene
	Persistent and diagnostic ciliary waveform abnormalities on high-speed video microscopy on multiple occasions
Children (1 month to 5 years)	
Two or more major PCD clinical criteria	
Plus at least one of the following	Diagnostic ciliary ultrastructure on electron micrographs
	Biallelic mutations in one PCD-associated gene
	Persistent and diagnostic ciliary waveform abnormalities on high-speed video microscopy on multiple occasions
Children 5–18 years of age and adults	
Two or more major PCD clinical criteria	
Plus at least one of the following	Nasal nitric oxide during plateau <77 mL/minute on two occasions, >2 months apart, with cystic fibrosis excluded
	Diagnostic ciliary ultrastructure on electron micrographs
	Biallelic mutations in one PCD-associated gene
	Persistent and diagnostic ciliary waveform abnormalities on high-speed video microscopy on multiple occasions
Major clinical criteria for PCD diagnosis	
Unexplained neonatal respiratory distress (at term birth) with lobar collapse and/or the need for respiratory support with CPAP and/or oxygen for >24 hours	
Any organ laterality defect—situs inversus totalis, situs ambiguous, or heterotaxy	
Daily, year-round wet cough starting in the first year of life or bronchiectasis on chest CT	
Daily, year-round nasal congestion starting in the first year of life or pansinusitis on sinus CT	

Confirming the diagnosis of PCD is a complex process, requiring a tertiary center with expertise and extended diagnostic capabilities. Such centers are often unavailable for most patients; thus, the diagnosis of KS is usually suspected through more commonly available testing. Children and adults with symptoms highly suspicious of KS should be referred for further testing. As recommended by the European Respiratory Society Task Force on PCD [7], current indications for further testing in adults and children are as follows: (1) patients found with situs inversus or heterotaxy, (2) children with unexplained cerebral ventriculomegaly, (3) siblings of patients, (4) neonates and infants with unexplained respiratory distress, (5) patients with chronic productive cough or bronchiectasis of unknown cause or severe upper respiratory morbidity, (6) males with immotile sperm, and (7) females with recurrent ectopic pregnancy.

There is no gold-standard test for PCD. Diagnosis is based on a combination of testing, including imaging, nasal nitric oxide levels, high-speed video microscopy, genetic testing, and transmission electron microscopy [8]. Analyzing all available testing is beyond the scope of this case report. As far as imaging is concerned, CT is the modality of choice [9]. High-resolution CT (HRCT) of the chest is very sensitive in detecting bronchiectases, especially in cases where they are more prominent in the middle and lower lobes. HRCT can also detect other causes of symptoms, such as emphysema and cystic fibrosis. CT of the sinuses can demonstrate chronic inflammation of nasal sinuses.

There is no curative treatment for KS. Patients with KS need a multi-specialist approach, as the disease impacts many systems [6]. Most treatment options aim to restore and maintain normal lung function, prevent hearing loss, and alleviate sinonasal symptoms. Treatments include aggressive use of antibiotics, nasal irrigation, physiotherapy, and physical exercise [7]. Patients need to be followed up closely by experienced specialists, with spirometry, imaging, and other testing.

## Conclusions

KS is a rare, chronic disease that has a negative impact on the quality of life of affected patients. This case report highlights the importance of suspecting KS in patients with suggestive features, regardless of their age, as early recognition is crucial in managing this disease. Imaging plays an important role in both diagnosis and monitoring. Management of these patients requires the collaboration of multiple specialists and long-term follow-up.
